# Fertility sparing strategies for pre- and peripubertal male cancer patients

**DOI:** 10.3332/ecancer.2020.1016

**Published:** 2020-02-27

**Authors:** Jan-Bernd Stukenborg, Christine Wyns

**Affiliations:** 1 NORDFERTIL Research Lab Stockholm, Childhood Cancer Research Unit, Department of Women’s and Children’s Health, Karolinska Institutet and Karolinska University Hospital, Solna, Sweden; 2 Cliniques Universitaires Saint-Luc, Université Catholique de Louvain, Brussels, Belgium

**Keywords:** testis, childhood cancer, infertility, fertility preservation, late effects, germ cells

## Abstract

Genetic parenthood following cancer therapy is considered to be a major factor of quality of life. Given the rising proportion of patients surviving cancer due to improved therapeutic protocols, it is an issue of growing importance. Hence, the efforts to preserve fertility have motivated researchers to develop options for the paediatric population facing fertility-threatening cancer therapies. In prepubertal boys who do not yet produce sperm, cryo-banking of testicular tissue containing spermatogonial stem cells (SSCs) is the only viable option for future fertility preservation. While proposed in a number of clinics worldwide, however, this strategy remains still experimental.

Transplanting the SSCs, or testicular tissue containing SSCs, back to the cured patient appears the most promising strategy. However, experiments performed with human testicular tissue in mice models reveal spermatogonial loss after transplantation, indicating the need for further optimisation of the transplantation procedure. The approach further poses the risk of reintroducing tumour cells back to the patient. In cases of haematological and blood-metastasising malignancies, *in vitro* generation of sperm combined with assisted reproductive technologies (ART), is the only possibility, avoiding reintroducing cancer cells. Although xenotransplantation would allow to recover sperm cells for ART being thus on the safe side with regard to cancer cells, the risk of infections with xeno-microbiological agents makes this option incompatible with clinical application. So far, offspring from *in vitro* matured sperm has only been achieved in mice. While human haploid germ cells, showing specific morphological features, expression of post-meiotic markers, as well as DNA and chromosome content, as well as fertilisation and development capacity, have been obtained by culturing spermatogonia or immature testicular tissue, the functionality of these cells still needs to be demonstrated. Despite the promising results obtained in recent years, further research is urgently warranted to establish a clinical tool offering these boys a fertility restoration option in the future. This mini-review will focus on current achievements and future challenges of fertility preservation in young boys and underscore the next steps required to translate experimental strategies into clinical practice.

## Introduction

Continuous efforts to improve oncological therapies have led to disease survival rates of more than 80% in the paediatric population [1–4]. A well-known side effect of cancer treatment is infertility in adulthood which is now a major concern for patients and their parents. However, the availability of comprehensive information on fertility preservation options prior to cancer therapies results in up to 74% acceptance rates for fertility preservation procedures [5]. To date, none of the strategies to reduce the gonadotoxicity of cancer treatments have proven successful in male patients [6]. In prepubertal boys, for whom spermatogenesis has not yet started, the conventional approach of sperm cryopreservation for fertility preservation is not an option as the testis only contains diploid germ cells, including the spermatogonial stem cells (SSCs). SSCs are established in the human testis during the first months after birth, when gonocytes migrate and attach to the basement membrane of the seminiferous cords. They are the starting point for continuous sperm production, they are present in the testis during the whole life, unless there is a congenital abnormality with germ cell aplasia or they are lost due to a disease or other event (e.g., medical treatment) [7]. As SSCs can only be identified based on their functionality, i.e., auto renewal and differentiation capacities, proxy markers for the spermatogonial population were used to evaluate their presence in the testicular tissue. Studies that analysed the presence of spermatogonia in prepubertal testicular tissue from patient that underwent a fertility preservation procedure showed that spermatogonia were present in 96% to 100% of samples [8, 9]. Hence, cryopreservation of testicular tissue samples—containing SSCs—has been proposed as the sole method to potentially preserve fertility. With regard to peripubertal boys, 20% in Tanner stage II of puberty already produce sperm [10]; therefore, cryopreservation of ejaculated semen from 12 years onwards or retrieval of testicular spermatozoa for those unable to provide a semen sample remains the priority. However, when no mature spermatozoa are collected, preserving the SSC via testicular tissue cryopreservation should also be considered for these patients.

## Prepubertal testicular tissue cryopreservation

Protocols to preserve human prepubertal testicular tissue allowing for survival and proliferation of SSCs as well as reinitiation of spermatogenesis have been reported for more than a decade [11–14]. So far, the superiority of one cryopreservation procedure over the other has not been demonstrated. Cryopreservation of immature testicular tissue is now proposed in a growing number of clinics. National and international networks have been established in Europe and the USA to enhance access and allow sample routing to facilities that meet the local regulations on human tissue and cell banking [7, 15, 16]. Reported complication rates due to the biopsy procedures are low [17].

At present, current recommendations for the procedure include: (i) performing the procedure within a multi-collaborative care pathway with trained care providers available to discuss the risk-benefit balance, (ii) taking the testicular sample before starting chemo- and or radiotherapy treatment (if possible), thereby ensuring the storage of SSCs with the best possible DNA integrity and (iii) to combine the procedure, whenever possible, with another procedure requiring anaesthesia and to restrict the sampling to one testis.

## Perspectives and challenges of fertility restoration strategies

As it stands, current investigations into the restoration of fertility using cryopreserved prepubertal testicular cells or tissue include *in vivo* approaches, such as autotransplantation of SSCs or immature testicular tissue, as well as *in vitro* development of mature sperm from SSCs. Here, we will briefly review the most recent achievements and challenges to be overcome before clinical implementation of the different strategies.

Autotransplantation of cryo-stored SSCs or immature testicular tissue can only be considered if there is no risk of cancer cell contamination of the testes. With regards to cell transplantation, attempts have been made to eliminate cancer cells from the testicular cell suspension through cell sorting techniques (for review see [18]) although further improvements and more powerful detection methods are required. Cell culture has also appeared useful to eliminate leukemic cells that were added to the testicular cell suspension although this study model may not be representative of cancer cell behaviour in tissues [19]. Transplantation of SSCs in seminiferous tubules was first described by Brinster and Avarbock in mice [20]. Generation of offspring in a number of other animal species has also been reported (for review, see [21]) with sperm obtained from transplanted SSCs in non-human primates resulting in viable embryos [22]. With the perspective of translating the technique to humans, methods to perform transplantation of cell suspensions in larger testes were evaluated. From these studies, ultrasound guided injection in the rete testis proved the most appropriate for human application [23]. *In vitro* propagation of SSCs will most likely be required before transplantation due to the scarcity of SSCs in the testis. Treatment related decrease of spermatogonia quantity reported in cancer patients [24, 25] as well as the poor colonisation efficiency of cell transplantation calculated in animal models [26, 27], also need to be taken in consideration. However, while *in vitro* propagation of human prepubertal SSCs was reported [28], safety issues linked to potential cell modification in culture as well as the functional proof that these cells are able to reinitiate sperm production, still need to be addressed.

Successful transplantation of freeze-thawed murine testicular tissue with birth of offspring was first described in 2002 [29], with subsequent reports in a number of other species (for review see [21]). Very recently, an important milestone was reached with the generation of offspring from transplanted immature testicular tissue in macaques [30]. Although yet to be translated to the clinic, it appears a likely frontrunner as the first fertility restoration option to enter pilot clinical trials. The procedure will however be restricted to patients with no risk of cancer recurrence, i.e., no possibility of neoplastic cells in the transplanted tissue {i.e., non-malignant conditions, e.g., haematological diseases requiring conditioning chemotherapy before bone marrow transplantation, such as sickle cell disease, thalassemia major or severe immune diseases non-responsive to immunotherapy (for review see [31])}. With regard to oncological patients, those with non-metastasising solid tumours could also come into consideration after tissue examination to exclude the presence of cancer cells in the tissue to be transplanted (providing that reliable techniques become available) and after fully informed patient-doctor decision and consent process. Currently, research is still ongoing to optimise tissue transplantation outcome as xenotransplantation experiments with human immature testicular tissue demonstrate a significant loss of spermatogonia, most likely due to hypoxia related to the absence of a vascular anastomosis between graft and host [18, 32, 33].

Options to restore fertility in patients with haematological or blood metastasising diseases are limited to strategies enabling the generation of sperm *in vitro* that may be used to fertilise eggs with intracytoplasmic sperm injection (for review see [34]).

The first studies demonstrating the potential of *in vitro* generated sperm to restore fertility were reported in the late 1990s when primary spermatocytes obtained from azoospermic patients were differentiated into haploid cells resulting in the birth of healthy infants after round spermatid injection in oocytes (ROSI) [35]. Other research groups revealed similar germ cell development using Vero cells to support the differentiation of spermatocytes *in vitro* [36]. More recently, *in vitro* generation of human haploid spermatids, able to develop into embryos after ROSI in murine oocytes, were obtained from SSCs of adult patients with cryptorchidism [37] and obstructive azoospermia [38].

The combination of functional somatic cells in direct contact with germ cells and the presence of a SSC microenvironment resembling the 3D-organisation of the seminiferous epithelium in situ have been highlighted as important factors to ensure survival and differentiation of male germ cells cultured *in vitro* (for review see [39]).

Hence, explant tissue or organotypic culture that maintains such 3D-organised microenvironment seems to be the most promising approach based on successful differentiation of murine SSCs into functional sperm *in vitro* [40] and the generation of human haploid germ cells from testicular tissue samples of cancer patients between 2 and 12 years of age [41, 42]. However, none of the reported studies demonstrated a system robust enough to differentiate human SSCs into functional sperm so far. Further research should focus on increasing knowledge on *in vivo* spermatogenesis to establish novel *in vitro* systems able to offer a safe, efficacious and efficient fertility preservation option for prepubertal cancer patients at risk of infertility.

## Conclusion

Today, testicular cell or tissue cryopreservation is the only option available for young patients unable to produce sperm, to preserve fertility with the hope of future parenthood. While cryopreservation protocols are available to preserve human testicular cells and tissue, techniques aimed at obtaining mature sperm from cryopreserved immature testicular tissue are still in development. Although encouraging results have been obtained, either *in vitro* or in animal models, such techniques are not currently appropriate for clinical practice. Therefore, cryopreservation programs should be performed within settings which allow for proper follow-up of patients in the event of reassessment of current practice. In addition, research on fertility restoration strategies should be performed, if possible, within multi-centre studies, to ensure high-quality research via standardised protocols. Health professionals, patients and their families should be informed about risks of treatments and the experimental nature of fertility preservation in prepubertal boys but also about research projects related to the field.

## Conflicts of interest

The authors declare that they have no conflict of interest.

## Funding

J.B.S acknowledges support from the Swedish Childhood Cancer Foundation (TJ2016-0093) and Swedish Research Council (2018-03094). C.W acknowledges support from the Fondation contre le cancer (FDC 2016-141), Fondation Salus Sanguinis and Fond National de la recherche scientifique de Belgique (Grants Télévie 7.4.572.09.F, 7.4616.11.F, 7.4596.13, 7.4554.14, 7.6511.16) for the experimental studies cited within this review.

## Authors’ contributions

Jan-Bernd Stukenborg and Christine Wyns contributed equally to this work.
